# Biosynthesis of Fluorescent β Subunits of C-Phycocyanin from *Spirulina subsalsa* in *Escherichia coli*, and Their Antioxidant Properties

**DOI:** 10.3390/molecules23061369

**Published:** 2018-06-06

**Authors:** Xian-Jun Wu, Hong Yang, Yu-Ting Chen, Ping-Ping Li

**Affiliations:** College of Biology and the Environment, Nanjing Forestry University, Nanjing 210037, China; yanghong0406108@163.com (H.Y.); takeiteasy0203@163.com (Y.-T.C.); ppli@njfu.edu.cn (P.-P.L.)

**Keywords:** phycocyanin, biosynthesis, antioxidant, *Spirulina*, gene expression, apo-CpcB

## Abstract

Phycocyanin, which covalently binds phycocyanobilin chromophores, is not only a candidate fluorescent probe for biological imaging, but also a potential antioxidative agent for healthcare. Herein, a plasmid harboring two cassettes was constructed, with *cpcB* from *Spirulina subsalsa* in one cassette and the fusion gene *cpcS::ho1::pcyA* in the other, and then expressed in *Escherichia coli*. PCB-CpcB(C-82), a fluorescent phycocyanin β subunit, was biosynthesized in *E. coli*, exhibiting an absorption maximum at 620 nm and fluorescence emission maximum at 640 nm. When *cpcS* was replaced by *cpcT*, PCB-CpcB(C-153), another fluorescent phycocyanin β subunit, was produced, exhibiting an absorption maximum at 590 nm and fluorescence emission maximum at 620 nm. These two fluorescent biliproteins showed stronger scavenging activity toward hydroxyl and DPPH free radicals than apo-CpcB. The IC_50_ values for hydroxyl radical scavenging by PCB-CpcB(C-82), PCB-CpcB(C-153), and apo-CpcB were 38.72 ± 2.48 µg/mL, 51.06 ± 6.74 µg/mL, and 81.82 ± 0.67 µg/mL, respectively, and the values for DPPH radical scavenging were 201.00 ± 5.86 µg/mL, 240.34 ± 4.03 µg/mL, and 352.93 ± 26.30 µg/mL, respectively. The comparative antioxidant capacities of the proteins were PCB-CpcB(C-82) > PCB-CpcB(C-153) > apo-CpcB, due to bilin binding. The two fluorescent biliproteins exhibited a significant effect on relieving the growth of *E. coli* cells injured by H_2_O_2_. The results of this study suggest that the fluorescent phycocyanin β subunits of *S. subsalsa* were reconstructed by one expression vector in *E. coli*, and could be developed as potential antioxidants.

## 1. Introduction

Phycobiliproteins are a type of light-harvesting biliprotein involved in photosynthesis in cyanobacteria and some eukaryotic alga [1,2,3]. Phycobiliproteins consist of at least two dissimilar types of peptides, with linear tetrapyrrole prosthetic groups covalently attached to the apoprotein via cysteine thioether linkages [4,5]. Phycobiliproteins exhibit unique absorbance and fluorescence properties and can be classified into three types, based on their absorbance maxima: phycoerythrin (PE, λ_max_: 540–570 nm), phycocyanin (PC, λ_max_: 610–620 nm), and allophycocyanin (APC, λ_max_: 650–655 nm) [6]. C-phycocyanin (CPC), one of the major phycobiliproteins, is composed of α and β subunits in the form of (αβ)_3_ or (αβ)_6_ aggregates. The α or β subunits of CPC can be assembled by apoproteins and chromophore groups in vivo or in vitro, with catalysis by the chromophore lyase. First, ferredoxin-dependent heme oxygenase (HO1) catalyzes the oxidative cleavage of the host heme to generate biliverdin [7], which is then reduced to phycocyanobilin (PCB) by phycocyanobilin:ferredoxin oxidoreductase (PcyA) [8]. Then, PCB is covalently attached to the apoprotein, which is catalyzed by specific lyases to generate fluorescent CPC. The lyases CpcE and CpcF are responsible for the attachment of PCB to the CPC α subunit [9]. The lyase CpcS catalyzes the attachment of PCB to the chromophore binding site Cys-β84 of CPC, while CpcT is able to bind PCB to Cys-β155 [10]. There are four genes that are considered to be essential for the formation of fluorescent CPC β subunits in *Escherichia coli*: *cpcB*, encoding apo-β-CPC (CpcB); *cpcS* or *cpcT*, encoding CpcS or CpcT, respectively; and *ho1* and *pcyA*, encoding HO1 and PcyA, respectively. Recently, a modular approach that requires only two DNA segments was reported [11]. The first segment is the *cpcB* gene from *Mastigocladus laminosus*, and the second is the fusion gene *cpcS::ho1::pcyA* or *cpcT::ho1::pcyA*, which was constructed as an end-to-end fusion of the respective genes from *Nostoc* sp. PCC 7120. The new synthetic pathway, despite requiring two plasmids, facilitates heterologous production of phycobiliproteins and the use of phycobiliproteins as fluorescent protein probes.

*Spirulina* is a genus of planktonic blue–green algae found in the alkaline water of volcanic lakes. Several in-vitro experiments have shown that *Spirulina* and its extracts, especially CPC, exhibit a variety of pharmacological effects, such as neuroprotective effects [12], hepatoprotective abilities [13], renoprotective effects [14], cardiovascular protective effects [15], and the elimination of cataracts [16], which is generally attributed to the antioxidant and free radical scavenging properties of CPC. Several studies have shown that the antioxidant activity is related to not only the bilin chromophore but also the apoprotein [17,18,19]. Some studies have been previously conducted to clone and express the relevant genes for producing α-CPC (apo-α-CPC and fluorescent α-CPC) in *E. coli* and evaluate their antioxidant activity [20,21]. Recently, recombinant apo-β-CPC has been shown to possess higher free radical scavenging activity than apo-α-CPC [22], and to be able to protect red blood cells from antioxidative damage [19]. The β-CPC subunits are considered to be highly effective antioxidants for pharmaceutical applications. However, to our knowledge, no report has been published on the antioxidant properties of recombinant fluorescent β-CPC.

In this study, the *cpcB* gene was cloned from *Spirulina subsalsa*. A modular approach with only one plasmid was employed to co-express the *cpcB* gene, as well as the fusion genes *cpcS::ho1::pcyA* or *cpcT::ho1::pcyA*, in *E. coli*, where the fluorescent biliproteins PCB-CpcB(C-82) and PCB-CpcB(C-153) were biosynthesized. Recombinant β-CPCs from *S. subsalsa* with a His tag were purified by Ni^2+^-chelated affinity chromatography, and showed similar spectral characteristics to previously reported fluorescent β-CPC submits [10]. Furthermore, the antioxidant activity of PCB-CpcB(C-82) and PCB-CpcB(C-153) was evaluated and compared with that of apo-CpcB. The result suggested that PCB-CpcB(C-82) and PCB-CpcB(C-153) hold great potential to be used as therapeutic agents for healthcare.

## 2. Results

### 2.1. Cloning of the cpcB Gene and Plasmid Construction

**Figure 1:**
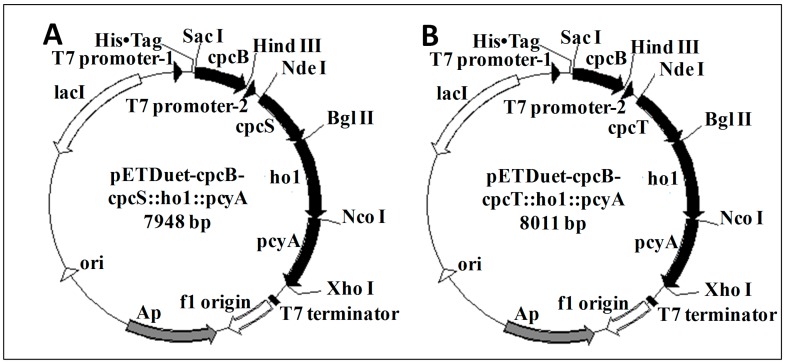
Schematic representation of the expression vectors pETDuet-cpcB-cpcS::*ho1*::*pcyA* (**A**) and pETDuet-cpcB-cpcT::*ho1*::*pcyA* (**B**).

Using designed primers, the 519-bp *cpcB* gene was amplified from *S. subsalsa* genomic DNA. A recombinant plasmid carrying the *cpcB* gene, namely, pETDuet-cpcB, was successfully constructed. Restriction enzyme digestion and DNA sequencing results showed that the *cpcB* gene was correctly inserted into the first multiple cloning sites of pETDuet, and fused in frame with the short hexahistidine tag sequence at the 5′ end. The final expression vectors, namely, pETDuet-cpcB-cpcS::*ho1*::*pcyA* and pETDuet-cpcB-cpcT::*ho1*::*pcyA*, were constructed by our methods. These vectors harbored pBR322-derived ColE1 replicons and *lacI* operator sequences for tight control of expression. Two cassettes of the final expression vectors carried *cpcB* and the genes encoding the fused enzymes under the control of separate T7 promoters (Figure 1). The *cpcB* gene followed by a His tag was in the first cassette for further purification. Upon analysis, the vectors exhibited the correct restriction enzyme patterns. Sequencing data showed the correct open reading frames in the correct orientations for *cpcB*, *cpcS*, *cpcT*, *ho1* and *pcyA* in the vector pETDuet.

### 2.2. Expression and Purification

**Figure 2:**
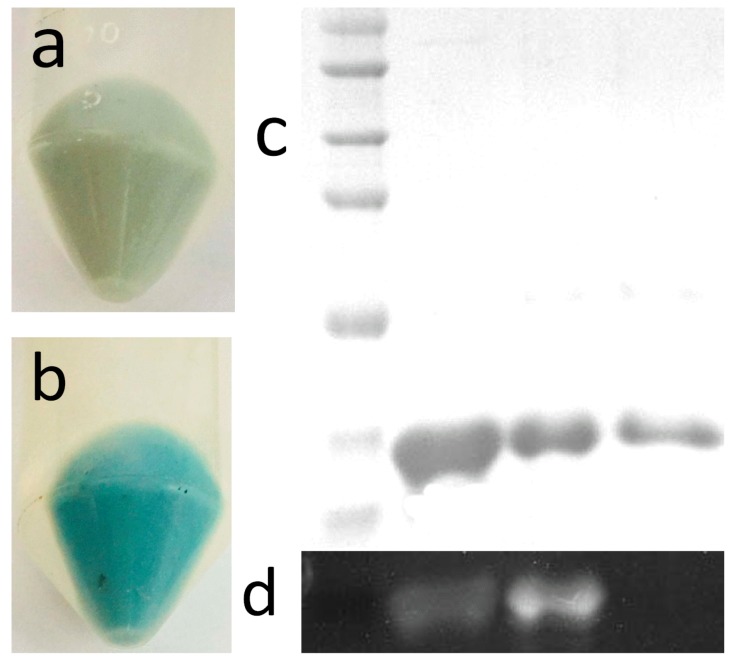
Color of the cell pellets of transformants harboring pETDuet-cpcB-cpcT::*ho1*::*pcyA* (**a**) or pETDuet-cpcB-cpcS::*ho1*::*pcyA* (**b**), and analysis of purified recombinant proteins by gradient SDS-PAGE (**c**) and chromoprotein Zn^2+^ electrophoresis (**d**). (**c**) Lane 1 shows standard protein makers—the protein bands correspond to 75, 60, 45, 35, 25, 20 and 15 kDa (from top to bottom); lane 2 shows PCB-CpcB(C-153); lane 3 shows PCB-CpcB(C-82); lane 4 shows apo-CpcB. (**d**) The chromoprotein Zn^2+^ electrophoresis results corresponding to lanes 1–4.

After induction with IPTG, the transformants harboring pETDuet-cpcB-cpcS::*ho1*::*pc*y*A* and pETDuet-cpcB-cpcT::*ho1*::*pcyA* exhibited a distinct blue-green color (Figure 2a,b), which implied that both PCB-CpcB(C-82) and PCB-CpcB(C-153) might be synthesized. The recombinant *E. coli* harboring pETDuet-cpcB as a control did not exhibit any color, due to a lack of phycobilin and lyases. Visualization of the three purified proteins with a purity ratio of 0.45 for PCB-CpcB(C-82) and 0.37 for PCB-CpcB(C-153) (Table 1) on a Coomassie blue-stained SDS-PAGE gel showed only one distinct band of 20 kDa, corresponding to the calculated molecular mass of apo-CpcB plus a His tag (Figure 2c). Covalent binding of the chromophore was confirmed upon exposure to Zn^2+^ and UV illumination, which resulted in a fluorescent emission (Figure 2d). The results confirmed the production of apo-CpcB and the chromophorylated derivatives of apo-CpcB in *E. coli*.

### 2.3. Absorbance and Fluorescence Spectrometry

**Figure 3:**
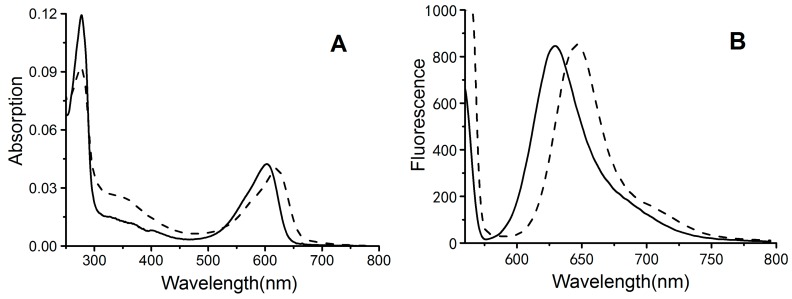
Absorption (**A**) and fluorescence (**B**) of the purified recombinant biliproteins PCB-CpcB(C-82) (dashed line) and PCB-CpcB(C-153) (solid line).

Recombinant biliproteins were further analyzed for spectral characteristics of absorption and emission. The results showed that both biliproteins exhibited unique absorption and fluorescence spectra in the visible region (Figure 3). PCB-CpcB(C-82) had an absorption maximum at approximately 621 nm and an emission maximum at approximately 646 nm, whereas PCB-CpcB(C-153) exhibited an absorption maximum at approximately 602 nm, with an emission maximum at approximately 629 nm (Table 1). The absorption and emission spectra of both biliproteins were similar to those of previously reported phycocyanins [10]. The recombinant proteins also exhibited a characteristic absorption peak at approximately 280 nm in the UV region, corresponding to the total protein concentration. The ratio of the absorption maxima of biliproteins (A_λmax_) to the absorption at 280 nm (A_280_) can reflect the approximate bilin-binding rate. The results showed that the bilin-binding rate of PCB-CpcB(C-82) (A_621_/A_280_) was 0.45, which was slightly higher than that of PCB-CpcB(C-153) (A_602_/A_280_ = 0.37) (Table 1), indicating that PCB-CpcB(C-82) may have higher antioxidant activity than PCB-CpcB(C-153), due to high bilin content.

### 2.4. Antioxidant Activity of Fluorescent Phycocyanin

**Figure 4:**
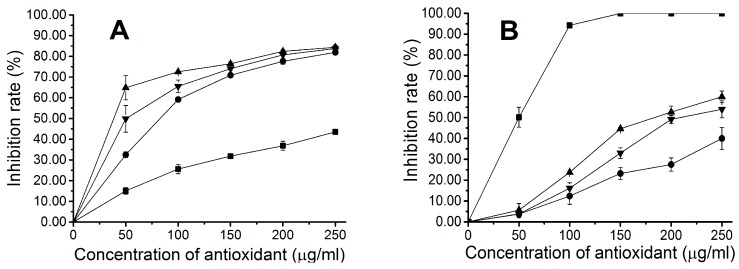
(**A**) Hydroxyl and (**B**) DPPH free radical scavenging activities of recombinant biliproteins; (▲) PCB-CpcB(C-82); (▼) PCB-CpcB(C-153); (●) apo-CpcB; (■) Mannitol (**A**) or ascorbic acid (**B**) (positive control).

Different concentrations of recombinant proteins were examined for antioxidant abilities by two different antioxidant assays, including assays of hydroxyl radical and DPPH radical scavenging activities. A deoxyribose assay was conducted to study the hydroxyl radical scavenging activity of apo-CpcB, PCB-CpcB(C-82), and PCB-CpcB(C-153), plus that of mannitol as a control. The result showed that all recombinant phycocyanins exhibited distinct hydroxyl radical scavenging activity, and the scavenging rates increased with increasing protein concentration (Figure 4). The recombinant phycocyanins exhibited higher hydroxyl radical scavenging activity than mannitol (Figure 4A). Furthermore, the scavenging activity of PCB-CpcB(C-82), with an IC_50_ value of 38.72 ± 2.48 µg/mL, was higher than that of PCB-CpcB(C-153), which has an IC_50_ value of 51.06 ± 6.74 µg/mL (Table 1). Apo-CpcB exhibited the lowest activity of the three recombinant proteins, with an IC_50_ value of 81.82 ± 0.67 µg/mL, but higher activity than previously reported apo-CpcB from *Spirulina platensis* [22].

The stable radical DPPH was used for determination of the antioxidant activity of apo-CpcB, PCB-CpcB(C-82), and PCB-CpcB(C-153), as well as that of ascorbic acid (Vc) as a control. The results showed that recombinant phycocyanins had DPPH free radical scavenging activity, and this activity increased with increasing protein concentration. However, the recombinant proteins exhibited significantly lower DPPH radical scavenging activity than ascorbic acid (Figure 4B). The IC_50_ values of PCB-CpcB(C-82), PCB-CpcB(C-153), and apo-CpcB were 201.00 ± 5.86 µg/mL, 240.34 ± 4.03 µg/mL, and 352.93 ± 26.30 µg/mL, respectively, which were 4~7 times higher than the IC_50_ value of ascorbic acid, which was 49.91 ± 0.32 µg/mL (Table 1).

The above results indicated that the three recombinant phycocyanins exhibited distinct scavenging capacities for both hydroxyl and DPPH radicals. The scavenging activity decreased in the following order: PCB-CpcB(C-82) > PCB-CpcB(C-153) > apo-CpcB. The antioxidant activity order of these phycocyanins significantly correlated with the rate of binding of bilin to the phycocyanin. Bilin and the apoprotein together contribute to the antioxidant properties of phycocyanin.

**Figure 5:**
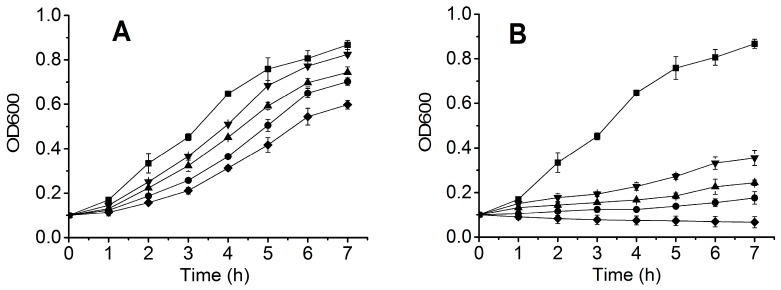
Inhibition of the cellular oxidative injury by the recombinant biliproteins (20 μg/mL). The samples were treated with 3 mM H_2_O_2_ (**A**) and 6 mM H_2_O_2_ (**B**); (▼) PCB-CpcB(C-82); (▲) PCB-CpcB(C-153); (●) apo-CpcB; (■) the control without H_2_O_2_ and the recombinant biliproteins; (◆) the control with H_2_O_2_, but without the recombinant biliproteins.

To detect the inhibition of the cellular oxidative injury by the recombinant biliproteins, the purified PCB-CpcB(C-82), PCB-CpcB(C-153), and apo-CpcB were respectively added into an *E. coli* culture system containing 3 and 6 mM H_2_O_2_ at a final concentration of 20 μg/mL. The results are shown in Figure 5. The addition of H_2_O_2_ made the growth of *E. coli* cells become slower compared to the control without H_2_O_2_, implying that *E. coli* cells were injured. The addition of the recombinant biliproteins significantly improves the survival of *E. coli* cells whether the concentration of H_2_O_2_ was 3 (Figure 5A) or 6 mM (Figure 5B). The inhibitory effect of the three recombinant phycocyanins on the oxidative injury of *E. coli* cells decreased in the following order: PCB-CpcB(C-82) > PCB-CpcB(C-153) > apo-CpcB. This is consistent with the effects of the hydroxyl and DPPH radical scavenging activities.

## 3. Discussion

Phycocyanins (PCs) are versatile biliproteins consisting of α and β subunits, and include fluorescent dyes [23], color additives [24], and antioxidant drugs [25]. Recombinant phycocyanins have received much attention due to their instability, the time-consuming purification process, the multimeric states of native phycocyanin, and especially because recombinant phycocyanins could potentially be used as genetically-encoded red and near-infrared fluorescent probes [26]. However, the multiple transformations involved in the process of phycocyanin biosynthesis are tedious and unpredictable. Wu et al. previously proposed a two-plasmid expression system with only two DNA segments to overcome this obstacle [11]. In this study, only one plasmid was needed to produce recombinant β subunits of C-phycocyanin, which emitted strong red fluorescence similar to that previously reported [11], further simplifying the biosynthesis of phycocyanin. Culture growth included only one resistant marker, which increased the stability of the transformants and reduced costs and antibiotic pollution in industrial applications. Guan et al. previously reported a different one-plasmid method, which employed a typical ribosomal binding site to construct an expression vector containing five genes [20]. Both one-plasmid methods could improve the rate of multigene transformation, which would be very important for the biosynthesis of phycocyanin in certain bacterial, fungal, plant, and animal cells.

Phycocyanin from *Spirulina* has attracted much attention because of the antioxidant properties and pharmacological effects of *Spirulina*. Some recombinant holo- and apophycocyanin α subunits have been reported to have higher antioxidant activities than the native phycocyanin [18,20]. The β subunits, which contain two bilin binding sites, are more complex, and might be more promising antioxidative agents than the α subunits [19]. To date, the antioxidant properties of recombinant fluorescent phycocyanin β subunits have not been reported, although those of recombinant apo-phycocyanin β subunits have been confirmed [22]. In this study, the antioxidant capacity of two recombinant fluorescent phycocyanin β subunits of *S. subsalsa* was assessed by radical scavenging assays, using hydroxyl and DPPH radicals, and by the inhibition effect of cellular oxidative injury. Our results indicated that the fluorescent phycocyanin β subunits had significantly better antioxidant potential than the apoprotein. The scavenging activity of the fluorescent phycocyanin C82-β subunit toward hydroxyl and DPPH radicals was 2.5 and 1.7 times higher than that of the apoprotein, respectively, and the scavenging activity of the fluorescent phycocyanin C153-β subunit was 1.5 and 1.4 times higher than that of the apoprotein, respectively. Meanwhile, the inhibition of the *E. coli* cells growth caused by H_2_O_2_ can be restored to some extent by the fluorescent phycocyanins β subunits, indicating that they have a good protective effect on the growth of *E. coli* cells injured by H_2_O_2_. As antioxidants, the fluorescent phycocyanin β subunits have a strong prospects for application in the food and medical industries. Moreover, Cherdkiatikul and Suwanwong reported that for both allophycocyanin and c-phycocyanin of *Spirulina*, the apo-β subunit possessed higher scavenging activity toward hydroxyl and peroxyl radicals than the apo-α subunit [22]. It is believed that the fluorescent phycocyanin β subunits have better antioxidant properties than the corresponding α subunits, but further confirmation is needed considering bilin content. In additional, more reactive oxygen or nitrogen species, such as superoxide anion, peroxynitrite, peroxyl radical, singlet oxygen, hydrogen peroxide, and hypochlorous acid [27] should be further considered for better assessment of the scavenging properties of the fluorescent phycocyanin β subunits.

The apoprotein of phycocyanin exhibited potent antioxidant activity, but bilin remained the major factor that influenced the antioxidative capability of phycocyanin. Our studies suggest that antioxidant activity increases significantly with chromophorylation of CpcB. Compared with the 153β subunit, the 82β subunit has a high chromophore binding rate (A_max_/A_280_ = 0.45 vs. 0.37), which contributes to the strong antioxidant properties of the 82β subunit (IC_50_ = 38.72 vs. 51.06 µg/mL or 201 vs. 240.34 µg/mL). Moreover, the two fluorescent β subunits also showed stronger inhibition of oxidative injury to *E. coli* cells than the apo-protein. It is speculated that the differences in the bilin binding rate among recombinant phycocyanin β subunits are responsible for the differences in antioxidant activities, but the antioxidant capabilities may also be associated with exposure levels of the chromophore embedded in the binding pocket. Nevertheless, it is believed that improved chromophore binding rates could significantly enhance the antioxidant capability of phycocyanin. The rate of bilin binding to the apoprotein is associated with various factors, such as expression and culture conditions, host strains and expression vectors, and characteristics of lyases and apoproteins [28,29]. Furthermore, in addition to the differences in amino acid sequences, homologs of the α or β subunits may exhibit distinct bilin-binding abilities. Therefore, it is necessary to assess the antioxidant capabilities of homologous α or β subunits of phycocyanin from different cyanobacteria, which, in combination with genetic modification, could further improve the antioxidant activity of phycocyanin.

To increase the bilin content of the recombinant phycocyanin β subunit, we attempted to simultaneously attach phycocyanobilin to two bilin binding sites of the β subunit in *E. coli*, in the presence of the lyases CpcS and CpcT. The fluorescent 82β subunit was obtained instead of the expected holo-β subunit, which may be due to the relatively low kinetic constants of CpcT compared with those of CpcS [30]. Future studies should be directed toward synthetizing the phycocyanin holo-β subunit in vivo or in vitro, and determining the antioxidative potential of this subunit. Notably, the fluorescent phycocyanin holo-β subunit should be an excellent candidate for antioxidant therapy, due to the increased bilin-binding capacity of this subunit.

## 4. Materials and Methods

### 4.1. Cyanobacterial Cultivation and DNA Extraction

Living alga *S. subsalsa* FACHB351 was obtained from the Freshwater Algae Culture Collection at the Institute of Hydrobiology, Chinese Academy of Sciences. The alga was cultured in SP medium at 25 °C. The light intensity was 50 μmol·m^−2^·s^−1^, with a 12 h light/12 h dark photoperiod cycle. The filaments were collected by centrifugation (5000 rpm) at the log phase. The *Spirulina* genomic DNA was extracted using TRIzol reagent (Invitrogen, Carlsbad, CA, United States) according to the manufacturer’s instructions.

### 4.2. Primer Design and PCR Amplification

The *cpcB* gene was amplified from *S. subsalsa* FACHB351 genomic DNA by polymerase chain reaction (PCR). The sequence for the primer design was obtained from the GenBank nucleotide sequence database (accession no. AY244667). The primers used to amplify the *cpcB* gene were as follows: forward primer, 5′-CAC GAG CTC TAT GTT TGA CGC ATT TAC AAG GGT TG-3′; reverse primer, 5′-TAT AAG CTT TTA GGC AAC AGC AGC AGC AGC G-3′. These primers were synthesized by Genscript. The resulting product was digested with the restriction enzymes Sac I and Hind III, and inserted into a Sac I- and Hind III-digested pUC57 cloning vector (Thermo Scientific^®^, San Jose, CA, USA), to generate the plasmid pUC57-cpcB, which was then sequenced to verify the integrity of cpcB.

### 4.3. Construction of the Expression Vector and Transformation

The *cpcB* gene of Spirulina was subcloned from pUC57-*cpcB* into the first expression cassette of an expression vector, namely, pETDuet (Novagen), yielding the plasmid pETDuet-*cpcB*. The plasmids co-expressing the phycobiliprotein lyase, heme oxygenase, and ferredoxin oxidoreductase (namely, pET30a-cpcS::*ho1*::*pcyA* and pET30a-cpcT::*ho1*::*pcyA*) were constructed as described by Wu et al. [11]. The constructs *cpcS*::*ho1*::*pcyA* and *cpcT*::*ho1*::*pcyA* were digested with Nde I and Xho I and individually inserted into the second expression cassette of the plasmid pETDuet-*cpcB*, yielding two expression plasmids, namely, pETDuet-cpcB-cpcS::*ho1*::*pcyA* and pETDuet-cpcB-cpcT::*ho1*::*pcyA*. The *cpcB* gene and the fused gene were located in two different expression cassettes of pETDuet. Each cassette contained a T7 promoter followed by a ribosome-binding site. The two expression plasmids were confirmed by restriction enzyme digestion analysis and DNA sequencing, then the plasmids were individually transformed into *E. coli* BL21 (DE3) cells, according to standard procedures. The plasmid pETDuet-*cpcB*, as a control, was also transformed into *E. coli* BL21 (DE3) cells. Transformants were selected with ampicillin.

### 4.4. Protein Expression and Purification

Transformed *E. coli* containing the constructed plasmids were cultured at 37 °C in Luria-Bertani (LB) medium, supplemented with ampicillin (100 μg/mL), until the optical density at 600 nm (OD_600_) was 0.5. Production of fluorescent biliprotein was induced by the addition of 1 mM isopropyl-β-D-thiogalactoside (IPTG). Cells were incubated with shaking at 150 rpm at 18 °C for 12 h in the dark. The cells were centrifuged at 9200× *g* for 5 min at 4 °C. Cell pellets were rinsed twice with distilled water and then re-suspended in ice-cold potassium phosphate buffer (KPB; 20 mM, pH 7.4) containing 0.5 M NaCl, and sonicated for 4 min at 200 W (JY92, SCIENTZ Biotechnology, Ningbo, China). The suspension was centrifuged via Ni^2+^-affinity chromatography on chelating Sepharose (Amersham Biosciences, Uppsala, Sweden) equilibrated with KPB containing 0.5 M NaCl. The proteins remaining on the column were eluted with the saline KPB, which also contained imidazole (0.5 M). After collection, the protein sample was dialyzed against the saline KPB overnight at 4 °C in the dark. The protein concentration was measured using the BCA Protein Assay Reagent Kit (Thermo Scientific, Waltham, MA, USA). The purified proteins were stored at −20 °C until further use.

### 4.5. Electrophoretic Analysis

The protein samples were analyzed by polyacrylamide gel electrophoresis in the presence of SDS. The SDS-PAGE gel was composed of a 10% separation gel and 5% stacking gel. The samples were boiled with 2× SDS sample buffer containing 30 mM β-mercaptoethanol for 5 min. The purified proteins were visualized by staining with Coomassie blue, and bilins were identified by Zn^2+^-induced fluorescence.

### 4.6. Spectral Analyses

Absorbance spectra were obtained on a Perkin Elmer Lambda 365 spectrophotometer. Fluorescence spectra were recorded at room temperature with a model LS 55 spectrofluorimeter (Perkin Elmer, Waltham, MA, USA). Excitation and emission slits were set at 10 nm for all measurements, with a scanning speed of 1200 nm/min.

### 4.7. Determination of Hydroxyl Radical Scavenging Activity

The hydroxyl radical scavenging ability of fluorescent phycocyanin was estimated by the deoxyribose assay. This method was performed as previously described by Bermejo et al. [31] and Cherdkiatikul and Suwanwong [22], with minor modifications. The reaction mixture contained 20 mM KPB (pH 7.4), 2.8 mM deoxyribose, 100 µM FeCl_3_, 100 µM EDTA, 1 mM H_2_O_2_, 100 µM ascorbic acid, and the test sample (50, 100, 150, 200, or 250 µg/mL). Solutions of FeCl_3_ and ascorbic acid were prepared immediately before use in de-aerated water and were mixed prior to addition into the reaction mixture. The reaction mixture (total of 500 µL) was incubated for 30 min at 37 °C. After incubation, the color was developed by adding 250 µL of thiobarbituric acid (1% *w*/*v* in 50 mM KOH) and 250 µL of trichloroacetic acid (2.8% *w*/*v* in deionized water), and heating the mixture in a boiling water bath for 15 min. The sample was then allowed to cool and diluted twofold with 20 mM KPB (pH 7.4). The absorbance was measured at 532 nm. KPB was the negative control. Mannitol, a classic hydroxyl radical scavenger, was used as a positive control. The hydroxyl radical scavenging activity was calculated using the following formula:

Hydroxyl radical scavenging activity (%) = [(A_0_ − A_1_)/A_0_] × 100

where A_0_ is the absorbance of the negative control, and A_1_ is the absorbance of the sample or the positive control. The IC_50_ (defined as the sample concentration at which 50% of the hydroxyl radicals were scavenged) was calculated for each sample.

### 4.8. Determination of DPPH Free Radical Scavenging Activity

The DPPH radical scavenging activity of fluorescent phycocyanin was tested according to the method of Xu et al. [32], with some modifications. The purified recombinant protein was diluted with KPB to 50, 100, 150, 200, and 250 µg/mL. Two milliliters of each dilution was added to a series of cuvettes following the addition of 500 µL of and ethanolic solution of DPPH (0.02%) and 1 mL of ethanol. The mixtures were gently mixed and incubated for 1 h at room temperature. The absorbance at 517 nm was measured after incubation. KPB was the negative control, and ascorbic acid was used as a positive control. DPPH radical scavenging activities were calculated by using the following formula:

DPPH radical scavenging activity (%) = [(A_0_ − A_1_ + A_2_)/A_0_] × 100

where A_0_ is the absorbance of the negative control; A_1_ is the absorbance of the test sample and the positive control; and A_2_ is the absorbance of the blank, without DPPH.

### 4.9. Inhibition of the Cellular Oxidative Injury by the Recombinant Biliproteins

Inhibition of the cellular oxidative injury by the recombinant biliproteins was performed as previously described by Yu et al. [21], with some modifications. *E. coli* BL21 (DE3) were grown overnight at 37 °C in 5 mL LB media. Aliquots of *E. coli* broths were separately transferred into five tubes pre-equipped with a 5 ml LB medium until OD600 = 0.1. The recombinant biliproteins were respectively added to the above three tubes at a final concentration of 20 μg/mL. A solution of 30% H_2_O_2_ was added to the above tubes, at a final concentration of 3 or 6 mM. The *E. coli* BL21 (DE3) cells without H_2_O_2_ and with H_2_O_2_ but without the recombinant phycocyanins were used as the control group. The *E. coli* BL21 (DE3) cells were cultured at 100 rpm for 7 h at 37 °C, and then the absorbance at 600 nm was determined every other hour. The growth curves of *E. coli* BL21 (DE3) cells were plotted to determine the inhibition of E. coli cells oxidative injury.

### 4.10. Statistics and Data Processing

All experiments were repeated at least three times. Data are presented as the mean ± SEM. The difference was considered statistically significant when *p* < 0.05. Statistical analysis was performed with SPSS (SPSS 17 for Windows, SPSS Inc., Chicago, IL, USA), using the IC_50_ values that were calculated.

## 5. Conclusions

We have cloned the *cpcB* gene encoding the phycocyanin β subunit from *S. subsalsa* FACHB351 and achieved the biosynthesis of two fluorescent phycocyanin β subunits, namely, PCB-CpcB(C-82) and PCB-CpcB(C-153), in *E. coli* with a one-plasmid method. The recombinant biliproteins exhibited significantly stronger scavenging activity toward hydroxyl and DPPH free radicals, and a stronger inhibitory effect on oxidative injury to *E. coli* cells than the recombinant apo-CpcB, due to covalent binding of the bilin chromophore to the apoprotein. The recombinant fluorescent phycocyanin β subunits possess high antioxidant capacities, and have potential applications in the food and pharmaceutical industries.

## Figures and Tables

**Table 1:** IC_50_ values representing the hydroxyl and DPPH free radical scavenging capacities of various proteins.^a^

Recombinant Proteins	Absorption	Fluorescence	Chromophore-Binding Rate (A_max_/A_280_)	IC_50_ Values (μg/mL)	
				Hydroxyl Radical Scavenging Activity	DPPH Free Radical Scavenging Activity
apo-CpcB	0	0	0	81.82 ± 0.67	352.93 ± 26.30
PCB-CpcB(C-82)	621	646	0.45	38.72 ± 2.48	201.00 ± 5.86
PCB-CpcB(C-153)	602	629	0.37	51.06 ± 6.74	240.34 ± 4.03
Ascorbic acid	－	－	－	－	49.91 ± 0.32
Mannitol	－	－	－	245.72 ± 9.43	－

^a^ The values are presented as the mean ± SEM.

## References

[1] Glazer A.N., Barber J. (1994). Adaptive variations in phycobilisome structure. Advances in Molecular & Cell Biology.

[2] Gantt B., Grabowski B., Cunningham F.X., Green B., Parson W. (2003). Antenna systems of red algae: Phycobilisomes with photosystem II and chlorophyll complexes with photosystem I. Light-Harvesting Antennas in Photosynthesis.

[3] Grossman A.R., Schaefer M.R., Chiang G.G., Collier J.L. (1993). The phycobilisome, a light-harvesting complex responsive to environmental conditions. Microbiol. Rev..

[4] Sidler W.A. (1994). The Molecular Biology of Cyanobacteria.

[5] Gao X., Wei T.D., Zhang N., Xie B.B., Su H.N., Zhang X.Y., Chen X.L., Zhou B.C., Wang Z.X., Wu J.W. (2012). Molecular insights into the terminal energy acceptor in cyanobacterial phycobilisome. Mol. Microbiol..

[6] Glazer A.N., Cohen Z. (1999). Phycobiliproteins. Chemicals from Microalgae.

[7] Beale S.I. (1993). Biosynthesis of phycobilins. Chem. Rev..

[8] Frankenberg N., Lagarias J.C., Kadish K.M., Smith K.M., Guilard R. (2003). Biosynthesis and Biological Functions of Bilins. The Porphyrin Handbook.

[9] Fairchild C.D., Zhao J., Zhou J., Colson S.E., Bryant D.A., Glazer A.N. (1992). Phycocyanin alpha-subunit phycocyanobilin lyase. Proc. Natl. Acad. Sci. USA.

[10] Zhao K.H., Su P., Tu J.M., Wang X., Liu H., Plöscher M., Eichacker L., Yang B., Zhou M., Scheer H. (2007). Phycobilin: Cystein-84 biliprotein lyase, a near-universal lyase for cysteine-84-binding sites in cyanobacterial phycobiliproteins. Proc. Natl. Acad. Sci. USA.

[11] Wu X.J., Chang K., Luo J., Zhou M., Scheer H., Zhao K.H. (2013). Modular Generation of Fluorescent Phycobiliproteins. Photochem. Photobiol. Sci..

[12] Penton-Rol G., Marin-Prida J., Pardo-Andreu G., Martinez-Sanchez G., Acosta-Medina E.F., Valdivia-Acosta A., Lagumersindez-Denis N., Rodriguez-Jimenez E., Llopiz-Arzuaga A., Lopez-Saura P.A. (2011). C-Phycocyanin is neuroprotective against global cerebral ischemia/reperfusion injury in gerbils. Brain Res. Bull..

[13] Vadiraja B.B., Gaikwad N.W., Madyastha K.M. (1998). Hepatoprotective effect of C-phycocyanin: Protection for carbon tetrachloride and R-(+)-pulegone-mediated hepatotoxicty in rats. Biochem. Biophys. Res. Commun..

[14] Fernandez-Rojas B., Medina-Campos O.N., Hernandez-Pando R., Negrette-Guzman M., Huerta-Yepez S., Pedraza-Chaverri J. (2014). C-phycocyanin prevents cisplatin-induced nephrotoxicity through inhibition of oxidative stress. Food Funct..

[15] Riss J., Decorde K., Sutra T., Delage M., Baccou J.C., Jouy N., Brune J.P., Oreal H., Cristol J.P., Rouanet J.M. (2007). Phycobiliprotein C-phycocyanin from Spirulina platensis is powerfully responsible for reducing oxidative stress and NADPH oxidase expression induced by an atherogenic diet in hamsters. J. Agric. Food Chem..

[16] Kumari R.P., Sivakumar J., Thankappan B., Anbarasu K. (2013). C-phycocyanin modulates selenite-induced cataractogenesis in rats. Biol. Trace Elem. Res..

[17] Lissi E., Pizarro M., Aspee A., Romay C. (2000). Kinetics of Phycocyanine Bilin Groups Destruction by Peroxyl Radicals. Free Radic. Biol. Med..

[18] Ge B., Qin S., Han L., Lin F., Ren Y. (2006). Antioxidant properties of recombinant allophycocyanin expressed in *Escherichia coli*. J. Photochem. Photobiol. B.

[19] Pleonsil P., Soogarun S., Suwanwong Y. (2013). Anti-oxidant activity of holo- and apo-c-phycocyanin and their protective effects on human erythrocytes. Int. J. Biol. Macromol..

[20] Guan X.Y., Zhang W.J., Zhang X.W., Li Y.X., Wang J.F., Lin H.Z., Tang X.X., Qin S. (2009). A potent anti-oxidant property: Fluorescent recombinant alpha-phycocyanin of *Spirulina*. J. Appl. Microbiol..

[21] Yu P., Li P., Chen X., Chao X. (2016). Combinatorial biosynthesis of Synechocystis PCC6803 phycocyanin holo-alpha-subunit (CpcA) in *Escherichia coli* and its activities. Appl. Microbiol. Biotechnol..

[22] Cherdkiatikul T., Suwanwong Y. (2014). Production of the alpha and beta Subunits of *Spirulina* Allophycocyanin and C-Phycocyanin in *Escherichia coli*: A Comparative Study of Their Antioxidant Activities. J. Biomol. Screen..

[23] Glazer A.N. (1994). Phycobiliproteins—A family of valuable, widely used fluorophores. J. Appl. Phycol..

[24] Rahman D.Y., Sarian F.D., van Wijk A., Martinez-Garcia M., van der Maarel M. (2017). Thermostable phycocyanin from the red microalga *Cyanidioschyzon merolae*, a new natural blue food colorant. J. Appl. Phycol..

[25] Romay C., Gonzalez R., Ledon N., Remirez D., Rimbau V. (2003). C-phycocyanin: A biliprotein with antioxidant, anti-inflammatory and neuroprotective effects. Curr. Protein Pept. Sci..

[26] Oliinyk O.S., Chernov K.G., Verkhusha V.V. (2017). Bacterial Phytochromes, Cyanobacteriochromes and Allophycocyanins as a Source of Near-Infrared Fluorescent Probes. Int. J. Mol. Sci..

[27] Trujillo J., Molinajijón E., Medinacampos O.N., Rodríguezmuñoz R., Reyes J.L., Loredo M.L., Barreraoviedo D., Pinzón E., Rodríguezrangel D.S., Pedrazachaverri J. (2015). Curcumin prevents cisplatin-induced decrease in the tight and adherens junctions: Relation to oxidative stress. Food Funct..

[28] Blot N., Wu X.J., Thomas J.C., Zhang J., Garczarek L., Bohm S., Tu J.M., Zhou M., Ploscher M., Eichacker L. (2009). Phycourobilin in trichromatic phycocyanin from oceanic cyanobacteria is formed post-translationally by a phycoerythrobilin lyase-Isomerase. J. Biol. Chem..

[29] Biswas A., Vasquez Y.M., Dragomani T.M., Kronfel M.L., Williams S.R., Alvey R.M., Bryant D.A., Schluchter W.M. (2010). Biosynthesis of cyanobacterial phycobiliproteins in *Escherichia coli*: Chromophorylation efficiency and specificity of all bilin lyases from *Synechococcus* sp. strain PCC 7002. Appl. Environ. Microbiol..

[30] Zhao K.H., Zhang J., Tu J.M., Bohm S., Ploscher M., Eichacker L., Bubenzer C., Scheer H., Wang X., Zhou M. (2007). Lyase activities of CpcS- and CpcT-like proteins from Nostoc PCC7120 and sequential reconstitution of binding sites of phycoerythrocyanin and phycocyanin beta-subunits. J. Biol. Chem..

[31] Bermejo P., Pinero E., Villar A.M. (2008). Iron-chelating ability and antioxidant properties of phycocyanin isolated from a protean extract of *Spirulina platensis*. Food Chem..

[32] Xu R., Li D., Peng J., Fang J., Zhang L., Liu L. (2016). Cloning, expression and antioxidant activity of a novel collagen from Pelodiscus sinensis. World J. Microbiol. Biotechnol..

